# Temporal Variations and Spatial Disparities in Public Sentiment Toward COVID-19 and Preventive Practices in the United States: Infodemiology Study of Tweets

**DOI:** 10.2196/31671

**Published:** 2021-12-30

**Authors:** Alexander Kahanek, Xinchen Yu, Lingzi Hong, Ana Cleveland, Jodi Philbrick

**Affiliations:** 1 College of Information University of North Texas Denton, TX United States

**Keywords:** COVID-19, preventive practices, temporal variations, spatial disparities, Twitter, public sentiment, socioeconomic factors

## Abstract

**Background:**

During the COVID-19 pandemic, US public health authorities and county, state, and federal governments recommended or ordered certain preventative practices, such as wearing masks, to reduce the spread of the disease. However, individuals had divergent reactions to these preventive practices.

**Objective:**

The purpose of this study was to understand the variations in public sentiment toward COVID-19 and the recommended or ordered preventive practices from the temporal and spatial perspectives, as well as how the variations in public sentiment are related to geographical and socioeconomic factors.

**Methods:**

The authors leveraged machine learning methods to investigate public sentiment polarity in COVID-19–related tweets from January 21, 2020 to June 12, 2020. The study measured the temporal variations and spatial disparities in public sentiment toward both general COVID-19 topics and preventive practices in the United States.

**Results:**

In the temporal analysis, we found a 4-stage pattern from high negative sentiment in the initial stage to decreasing and low negative sentiment in the second and third stages, to the rebound and increase in negative sentiment in the last stage. We also identified that public sentiment to preventive practices was significantly different in urban and rural areas, while poverty rate and unemployment rate were positively associated with negative sentiment to COVID-19 issues.

**Conclusions:**

The differences between public sentiment toward COVID-19 and the preventive practices imply that actions need to be taken to manage the initial and rebound stages in future pandemics. The urban and rural differences should be considered in terms of the communication strategies and decision making during a pandemic. This research also presents a framework to investigate time-sensitive public sentiment at the county and state levels, which could guide local and state governments and regional communities in making decisions and developing policies in crises.

## Introduction

### Background

The COVID-19 pandemic has had worldwide economic and mortality impacts, with more than 118 million confirmed cases and over 2.6 million deaths globally as of March 12, 2021 [1]. Since the initial outbreak of COVID-19, many public health professionals and authoritative organizations, such as the Centers for Disease Control and Prevention (CDC) and the World Health Organization, have recommended that people change their fundamental behaviors of daily life to prevent the virus from spreading, for example, wearing masks, social distancing, and restricting travel [2]. However, the effectiveness of these measures in reducing the spread hinges on compliance by the public. The level of compliance varies among citizens in following the suggested practices. In the United States, there are divergent opinions about the preventive practices, which have existed from the onset of the CDC guidelines.

### Prior Work

It is critical to gauge public sentiment and responses to the preventive practices for effective communication strategies, decisions, and policies, as disparities in practices may affect the spread of the disease and delay society’s recovery from the pandemic. Social media has been widely adopted by people to acquire information and share opinions in crises, which provides time-sensitive opportunities for governments and public institutions to understand public opinions. Social media data have been used as crowd sources of information to understand citizens’ issues of concern [3,4], response to policies [5,6], and emotional consequences [7] in crises. Several recent studies have used Twitter and Facebook data for closer-to-real-time infodemiology studies, for example, to analyze emotions concerning the lockdown [8] and reopening [9] and to understand COVID-19 discussions and the associated sentiments [10]. However, these studies usually rely on an implicit assumption that strategies based on the understanding of the whole society at a time or during a time range work for all. Some studies have investigated the evolvement of public responses as the crisis unfolded, for example, the content analysis of crisis-related tweets before, during, and after the crisis [11]; temporal variations of public sentiment toward COVID-19 in China [12]; and changes in risk perception of COVID-19 in the United States in the early stage of the pandemic [13]. Several studies have examined the spatial differences. For example, Ntompras et al [14] conducted comparisons of the content of Twitter posts related to the COVID-19 pandemic across nations. They found several topics were triggered by local events, which implies that social media data can act as political, economic, and social monitoring in pandemics. Cuomo et al [15] performed a more granular analysis and investigated the longitudinal and geospatial relationships between volumes of self-reporting COVID-19 cases and elevated risks of virus spreading in the United States at the county level. Similar studies have found geolocated tweets on COVID-19 symptoms, concerns, and experiences are indicative of officially reported COVID-19 cases at the county level in the United States [16] and volumes of misinformation are related to increased COVID-19 cases at the state and county level in the United States [17]. Currently, few have investigated the temporal variations in public sentiment in a high geospatial resolution. Hou et al [18] found that mobility behaviors differ in communities during COVID-19, which could be related to various socioeconomic and cultural factors. Schmelz [19] conducted a survey study in Germany and found that people with different levels of trust in the government or with different political identities may have varying reactions in their response to government policies during COVID-19. These studies suggest it is important to consider the heterogeneity of the population in public health decision making. Methods on the time-sensitive understanding of a crisis with social media data have seldomly considered the geographical disparities and the associated socioeconomic factors. This study aimed to address this gap by proposing a social media data analysis framework for a longitudinal investigation of US public sentiment about COVID-19 and the preventive practices on different spatial scales.

### Goal of This Study

The focus of the study was to identify the variations in public sentiment toward COVID-19 and the preventive practices from the temporal and spatial perspectives and to investigate how the variations are related to geographical and socioeconomic factors in the United States. Specifically, we analyzed discussions of COVID-19 on Twitter in the United States to answer the following questions:

1. Research question 1: Are there temporal variations in public sentiment toward overall COVID-19 issues and preventive practices?
2. Research question 2: Are there spatial disparities in public sentiment toward overall COVID-19 issues and preventive practices?
3. Research question 3: What geographic factors may be related to the differences in public sentiment toward COVID-19 issues and preventive practices?
4. Research question 4: What socioeconomic factors may be related to the differences in public sentiment toward COVID-19 issues and preventive practices?

Exploring these 4 questions could offer rich insight into public sentiment about COVID-19 issues and preventive practices, with fine temporal and spatial granularity. This allows policy makers to explicitly consider these variations in developing communication strategies or adjusting enforcement policies for efficient coordination in pandemics or crises like COVID-19. This study sets the groundwork for analyzing, comparing, and potentially predicting public sentiment in future crises.

## Methods

**Figure 1:**
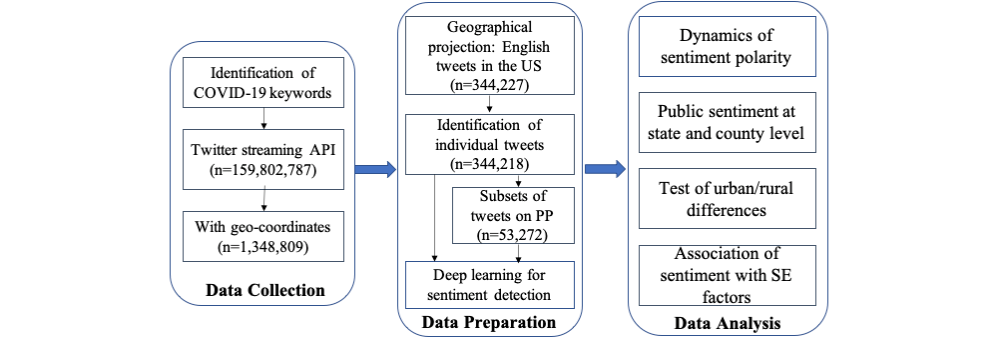
Data analysis framework. API: application programming interface; PP: preventive practices; SE: socioeconomic.

The method was composed of 3 parts (Figure 1): data collection, data preparation, and data analysis. In this study, we collected and analyzed Twitter data on COVID-19 and the preventive practices.

### Data Collection

COVID-19 cases were first reported in Wuhan, China in late December 2019. The disease was fast-spreading and led to increasing infections and deaths globally. Starting in January 2020, other countries started to report confirmed cases of COVID-19. To retrieve online discussions on COVID-19, we collected a Twitter data set with about 160,000,000 tweets containing COVID-19–related keywords starting from January 21, 2020. The list of keywords includes “Coronavirus,” “Corona,” “CDC,” “Covid19,” “Covid19,” “Sarscov2,” “pandemic,” “epidemic,” and their variants [20]. The data were collected using Python through the Twitter’s streaming application programming interface (API). The Twitter streaming API returns 1% of the total Twitter volume, with multilingual tweets posted from around the world. As we were focused on the public sentiment in the United States, only tweets in English were kept.

### Ethics Statement

In compliance with Twitter policy, we removed identifiers from the data before analysis to avoid potential profiling or targeting of individuals. We only present aggregated analyses. To support reproducibility, the tweet IDs, processing code, and intermediate results will be available upon request to the corresponding author.

### Data Preparation

Data preparation was composed of 4 parts (Figure 1): the geographical projection of tweets, the identification of posts from individual users, the subsetting of topics on preventative practices, and sentiment detection. All the data preparation was implemented with Python.

#### Geographical Projection

The collected tweets only satisfied the condition of semantic relevance to COVID-19, of which some had embedded geolocations, such as a point location, a bounding box defined with geographical coordinates, or user-entered location tags. Although many tweets contain location tags, such tags often vary in geographical scale or do not refer to real locations. Therefore, only tweets with geographical coordinates, either as a point location or a bounding box, were used. We used the GeoPandas package in Python for all geographical data processing. After calculating the center of the bounding box, they were projected to the coordinate system of the Shapefile map of the United States [21] and then matched with the geographical units at the county and state levels. If the location of a tweet fell in a county, we assigned the tweet with the associated county and state, together with the aggregated socioeconomic information from the US Census Bureau [22]. In addition, we used the urban/rural map Shapefile to identify whether a tweet was posted from an urban or rural area [23]. Urban areas included “Urbanized Areas of 50,000 or more people and Urban Clusters of at least 2,500 and less than 50,000 people” [24]. Other areas were classified as rural. After geographical projection and filtering for tweets only posted in the English language that were located within the United States, there was a total of 344,227 tweets.

#### Identification of Posts From Individual Users

Different types of users are on Twitter, including media outlets, accounts of government authorities and organizations, social bots, and individual accounts. The quality of data and the generated insights may be impacted by the activities of bots and official accounts [7]. The first step of geographical projection left tweets that were highly probable to be from individual users. To assure that the tweets used for analysis were mainly from individual users, we applied the traditional approach by checking the social relations of the authors, assuming that media outlets and bots usually have a high ratio between their followers and friends:




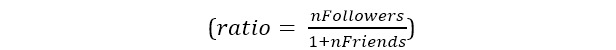




Specifically, we identified users whose numbers of followers and followees were 2 SDs larger than the average values as nonpersons [25]. We found 9 tweets by media outlets or bots. The result confirmed the filtered geolocated tweets to be mainly from individual users. After filtering for individual users, the COVID-19 data set included 344,218 tweets.

#### Tweets on COVID-19 Preventive Practices

We were specifically interested in the public sentiment toward COVID-19 prevention practices, as people's compliance to these practices highly affect the spread of the disease. To identify the potential keywords that describe COVID-19 prevention practices, we collected all the guidelines released by the CDC [2]. Three graduate research assistants read through the documents and identified keywords and phrases that were relevant to the preventive behaviors for reducing the spread of the disease. Specifically, 4 categories of practices were collected, including physical or social distancing, personal protective equipment (PPE), disinfection, and other. Physical or social distancing included social distancing, social distance, physical distance, 6-feet, stay-at-home, school isolation, isolation, stay home, avoid touching. PPE included mask, covering, face shield, wear a mask, surgical mask, N95 respirator, wearing gloves, face shields, facial covering, skin protection, eye protection, PPE. Disinfection included wash hands, hand sanitizer, disinfect, clean, detergent, handwash, hand hygiene, prevention hygiene, sprays, concentrates, wipes, routine cleaning, bleach solution. Others included test, business closure.

These keywords and phrases were used to identify if a tweet was about COVID-19 preventive practices and the category of preventive practices. As the language used in social media posts may have syntax or typographical errors, using the formal keywords and phrases from CDC guidelines may affect the recall of tweets on preventive practices. Therefore, we applied token normalization for both tweets and the keywords and phrases. After the normalization of each tweet, we checked if any tokens in a tweet matched the normalized keywords or phrases. Tweets containing these keywords or phrases were aggregated to form the subset on preventive practices (shortened to CDC subset in the following analysis), which had a total of 53,272 tweets. Based on the tokens, the tweets were further categorized as discussions on 1 of the 4 categories. The top keywords found in the COVID-19 data set were mask, stay home, social distancing, test, and PPE. These individual keywords had more than 8000 occurrences.

#### Sentiment Detection

We used a pretrained deep learning model, FLAIR, to detect the sentiment contained in each tweet [26]. The model was constructed with the recurrent neural network architecture, which enables the capture of semantic and syntactic information of words and the surrounding context for the prediction of the sentiment of input text. As the model was designed to capture different meanings for polysemous words and handle rare and misspelled words with ease, it works well for a Twitter corpus, where words are often misspelled and have ambiguous meanings. The model had state-of-the-art performance in sentiment classification, with an accuracy of 89.5% and an F1 score of 0.89 on a separate data set [27].

**Figure 2:**
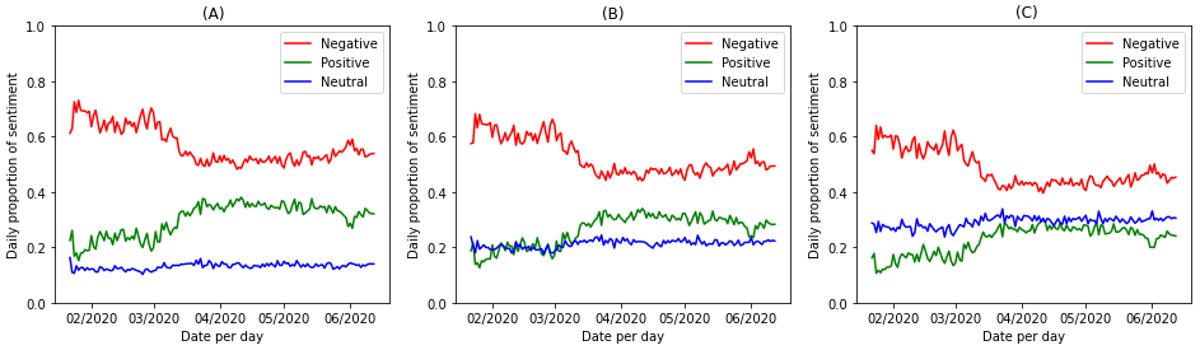
The daily proportion of sentiment in the COVID-19 data set when neutral tweets were detected with different confidence thresholds: (A) 0.8, (B) 0.9, (C) 0.95.

For each input tweet, the output was a sentiment of 1 of 2 categories: positive or negative with the associated confidence of the model’s prediction. However, not all tweets include the expression of sentiment. In fact, about 25% of crisis-related Twitter data do not contain subjective information [28]. Tweets with a low confidence to a sentiment category are probable to be neutral or objective. As each sentiment category has a confidence between 0 and 1, we explored 3 thresholds (0.8, 0.9, and 0.95) to understand whether the choice of thresholds would affect the temporal variations of sentiment. Figure 2 shows the ratio of COVID-19 tweets that are positive, negative, and neutral on a daily level, when the confidence thresholds of 0.8, 0.9, and 0.95 were used to define neutral tweets. We found that the choice of threshold would not significantly impact the temporal patterns of positive or negative sentiment in the COVID-19 data set. To obtain more samples for analysis, we chose the confidence level of 0.8 and considered tweets with a confidence level of positive or negative less than 0.8 as neutral.

#### Summary of the Data Set

Table 1 shows the summary statistics of the COVID-19 data set and the CDC subset for analysis. Both data sets have tweets from 50 states plus Washington DC and other territories of the United States in the time range from January 21, 2020 to June 12, 2020.

**Table 1:** Descriptive summary of the COVID-19 data set and the Centers for Disease Control and Prevention (CDC) subset.

Characteristics	COVID-19	CDC
Tweets, n	344,218	53,272
Negative sentiment, n (%)	195,166 (56.7)	32,408 (60.8)
Positive sentiment, n (%)	103,698 (30.1)	13,411 (25.2)
Neutral sentiment, n (%)	45,354 (13.2)	7453 (14.0)
Tweets per day, mean (SD)	2424 (1488.96)	375 (294.55)
Tweets per week, mean (SD)	16,391 (6935.53)	6935 (1529.67)

### Analysis of Temporal Variations and Spatial Disparities

We conducted temporal analysis to answer research question 1. First, we computed the ratio of tweets with positive, negative, or neutral sentiment separately in the granularity of a day for the COVID-19 data set and the CDC subset and weekly for each category of the preventive practices (ie, physical or social distancing, PPE, disinfection, and others). The time series of public sentiment were analyzed with an algorithm that helped to detect the turning points when the sentiment patterns started to change. The turning points and the nearby dates were investigated to explore what events might be related to the significant changes in public sentiment polarity.

The enforcement policies issued by state and local governments and the dates of intervention could be different in the United States. The enforcement may trigger changes in public sentiment to preventive practices [29]. We conducted county- and state-level analyses to examine the spatial disparities. We aggregated the tweets by states and generated sentiment polarity maps for the COVID-19 data set and CDC subset. Further, 4 representative states were analyzed to investigate the dynamics of public sentiment at a finer spatial granularity, which enabled analysis of whether the changes in public sentiment related to state-level events or policies.

The variations in public sentiment in different regions could be related to the heterogeneity of the population, as existing studies have shown the response behaviors in COVID-19 are related to cultural, socioeconomic, and political factors [19,29,30]. Two types of analysis were implemented to answer research questions 3 and 4. The analysis was conducted at the aggregated county level. We did not investigate aggregation at a finer spatial granularity, such as by census tracts or census block groups, due to the sparsity of tweets with geolocations. Using a smaller geographical unit means fewer tweet samples in each unit, which could be easily affected by sentiment detection errors.

First, we compared the public sentiment polarity in the urban and rural areas of counties to answer research question 3. For each county, we obtained the ratio of tweets with positive, negative, and neutral sentiments separately for urban and rural areas. We ran a *t* test between the sentiment polarity in the urban and rural areas of counties to find out if the urban/rural factor would explain the variances in the public sentiment to COVID-19 and preventive practices. Second, we examined if the sentiment polarity to COVID-19 and preventive practices were statistically related to the socioeconomic factors for research question 4. The socioeconomic information was obtained from the *U.S. Census Bureau Indicators of the 2017 American Community Survey 5-year Estimate* [22].

## Results

### Temporal Variations in Public Sentiment Toward COVID-19 and Preventive Practices

**Figure 3:**
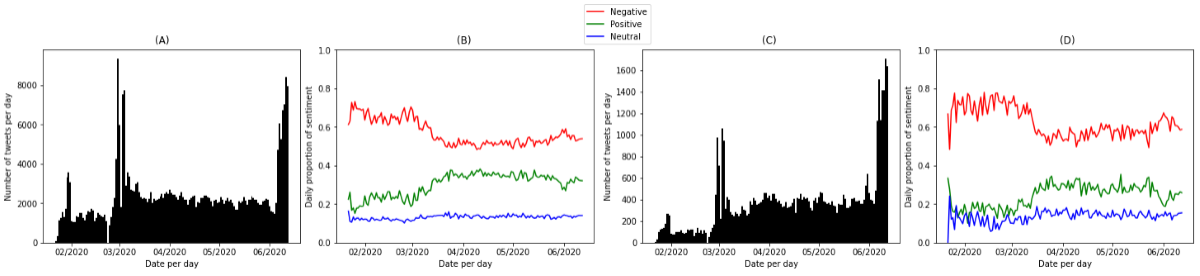
The numbers of tweets and proportions of sentiment per day in (A) and (B), respectively, the COVID-19 data set and (C) and (D), respectively, the Centers for Disease Control and Prevention (CDC) subset.

Figure 3 presents the visualization of volumes and sentiment polarity separately for tweets in the COVID-19 data set and the CDC subset. Tweets on preventive practices represented about 15.5% (53,272/344,218) of COVID-19 tweets. The 2 timelines on volumes showed a common pattern and had 2 large spikes at similar time points: one in the beginning of March 2020 and another in the middle of June 2020. The timing of these 2 spikes corresponded to the turning points when public sentiment polarity started to change. Since the beginning of March 2020, the negative sentiment about COVID-19 issues and preventive practices started to decrease, although the second spike in June 2020 was associated with increasing negative sentiment in both data sets.

**Figure 4:**
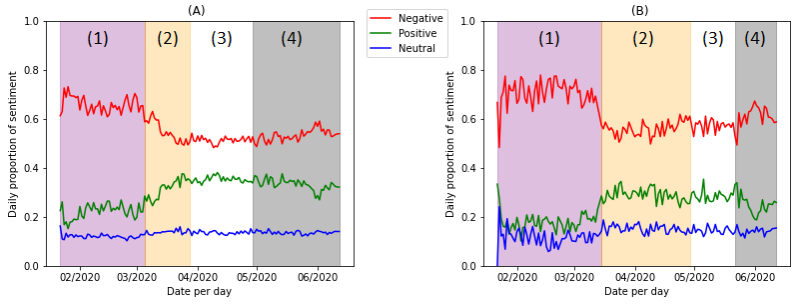
Stage splitting for the (A) COVID-19 data set and (B) Centers for Disease Control and Prevention (CDC) subset.

The daily proportion of neutral tweets had little variation over the studied time range; the time series of positive sentiment was almost mirrored to that of the negative sentiment. Therefore, we focused on the analysis of the negative sentiment. Figure 4 presents the visualization of the 4 stages indicated by 4 different colors. The turning points about COVID-19 were March 6, 2020; March 29, 2020; and April 30, 2020. Table 2 and Table 3 show the summary statistics of the 4 stages for the COVID-19 data set and the CDC subset.

The dynamics of negative sentiment in the COVID-19 data set and CDC subset shared similar patterns, except that the timing of turning points varied. The COVID-19 data set and the CDC subset both had a high proportion of negative sentiment in Stage 1. The mean daily proportions of negative tweets were 66.6% (59,805/89,757) in the COVID-19 data set and 70.7% (8107/11,475) in the CDC subset. In Stage 2, there was a consistent decline in the negative sentiment in both the COVID-19 data set and the CDC subset, although the turning point of the COVID-19 time series (March 5, 2020) came earlier than that of the CDC subset (March 15, 2020). After a certain amount of time, the decreasing trend stopped and reached another turning point. In Stage 3, the negative proportion remained stable in the COVID-19 data set. The average negative proportion (37,350/72,849, 51.3%) was lower than in Stage 1 (59,805/89,757, 66.6%) and Stage 2 (32,062/58,010, 55.3%) in the COVID-19 data set. Comparatively, there were more variations in the negative sentiment in the CDC subset. People showed increasing negative sentiment toward preventive practices in Stage 3 (4945/8610, 57.4%) and Stage 4 (9937/16,328, 60.9%) after Stage 2 (9419/16,859, 55.9%). There was also an increasing trend in negative sentiment toward general COVID-19 issues in Stage 4 (65,954/123,602, 53.4%). In all stages, the sentiment polarities in the CDC subset were higher than those in the COVID-19 data set.

There were similar trends by categories. The negative sentiment polarities were the highest at the beginning of COVID-19 from January. Then, the proportion of negative tweets decreased until later May 2020 when negative sentiment started to rebound. There were also noticeable differences. For example, the sentiment for the disinfection topic had a small spike in April 2020, which could be related to the criticism of “disinfectant injection.” Overall, public sentiment to the PPE topic (11,157/19,640, 56.8%) was more negative than to physical or social distancing (10,684/19,466, 54.9%) and disinfection (1706/3061, 55.8%).

**Figure 5:**
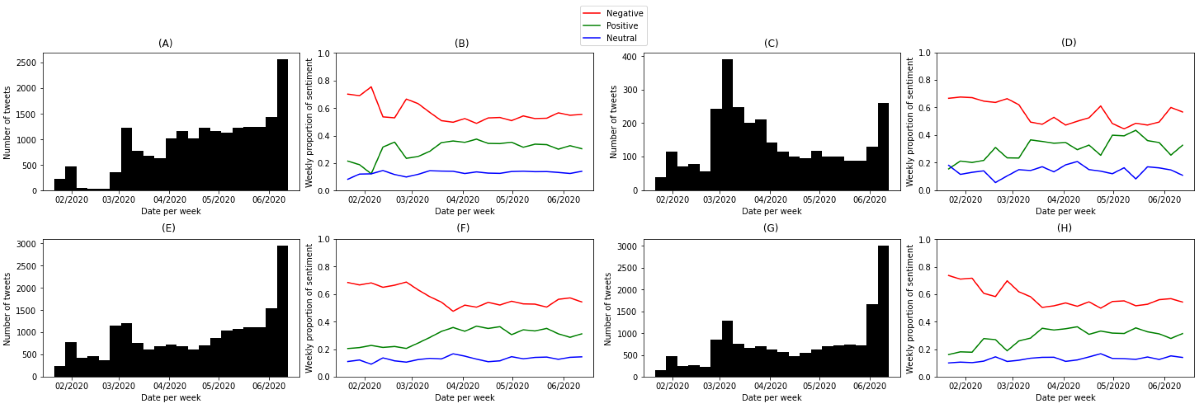
The number of tweets and proportion of sentiment per week in the Centers for Disease Control and Prevention (CDC) subset, split by themes: (A) and (B), respectively, physical or social distancing; (C) and (D), respectively, disinfection; (E) and (F), respectively, personal protective equipment; (G) and (H), respectively, others.

To further investigate public sentiment toward different categories of preventive practices, we generated the timelines of public sentiment polarity separately for tweets in the 4 categories (ie, 19,640 tweets on PPE, 19,466 tweets on physical or social distancing, 3061 tweets on disinfection, and 16,425 tweets on other measurements). As we investigated more detailed granularity, there were fewer representative samples at the daily level, which led us to adjust the aggregation from the daily level to the weekly level. Figure 5 shows the visualization of weekly volumes and sentiment polarity of tweets in the 4 categories of preventive practices.

**Table 2:** Summary statistics for the 4 stages of the COVID-19 data set.

Stage	Date range	Volume	Negative sentiment	Positive sentiment	Neutral sentiment
1	January 21, 2020 to March 5, 2020	89,757	0.6663	0.2140	0.1197
2	March 6, 2020 to March 28, 2020	58,010	0.5527	0.3102	0.1371
3	March 29, 2020 to April 29, 2020	72,849	0.5127	0.3521	0.1352
4	April 30, 2020 to June 12, 2020	123,602	0.5336	0.3304	0.1360
Total	January 21, 2020 to June 12, 2020	344,218	0.5669	0.3013	0.1318

**Table 3:** Summary statistics for the 4 stages of the Centers for Disease Control and Prevention (CDC) subset.

Stage	Date range	Volume	Negative sentiment	Positive sentiment	Neutral sentiment
1	January 21, 2020 to March 5, 2020	11,475	0.7065	0.1780	0.1155
2	March 6, 2020 to March 28, 2020	16,859	0.5587	0.2895	0.1518
3	March 29, 2020 to April 29, 2020	8610	0.5743	0.2825	0.1432
4	April 30, 2020 to June 12, 2020	16,328	0.6086	0.2484	0.1430
Total	January 21, 2020 to June 12, 2020	53,272	0.6084	0.2517	0.1399

### Spatial Disparities at the State Level

**Figure 6:**
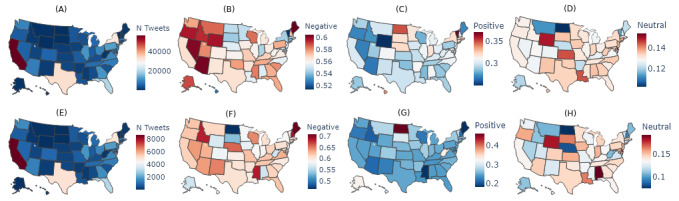
The number of tweets and proportion of sentiment for each state across the entire timeline: (A) count of tweets in the COVID-19 data set; (B) proportion of negative sentiment in the COVID-19 data set; (C) proportion of positive sentiment in the COVID-19 data set; (D) proportion of neutral sentiment in the COVID-19 data set; (E) count of tweets in the Centers for Disease Control and Prevention (CDC) subset; (F) proportion of negative sentiment in the CDC subset; (G) proportion of positive sentiment in the CDC subset; (H) proportion of neutral sentiment in the CDC subset.

Figure 6 shows the number of tweets on COVID-19 and preventive practices at the state level in the United States; 4 states had the largest number of tweets on COVID-19 and preventive practices: California, New York, Texas, and Florida. These 4 states are the most populated states in the United States according to the US Census Bureau [22]. The counts of tweets, per state, were proportionally similar between the COVID-19 and CDC data sets, ensuring that changes in sentiment between the 2 data sets were not due to geographical sampling differences. The sentiment polarity map of the COVID-19 data set showed that negative sentiment was highest in Maine and some of the states in the Pacific Western area including Arizona, Nevada, Wyoming, Oregon, and Idaho. More research needs to be done to investigate why the negative sentiment presented such a geographic pattern. On the other hand, states with the most negative sentiment on CDC were geographically dispersed. The top 3 states included Maine, New Hampshire, and Mississippi.

**Figure 7:**
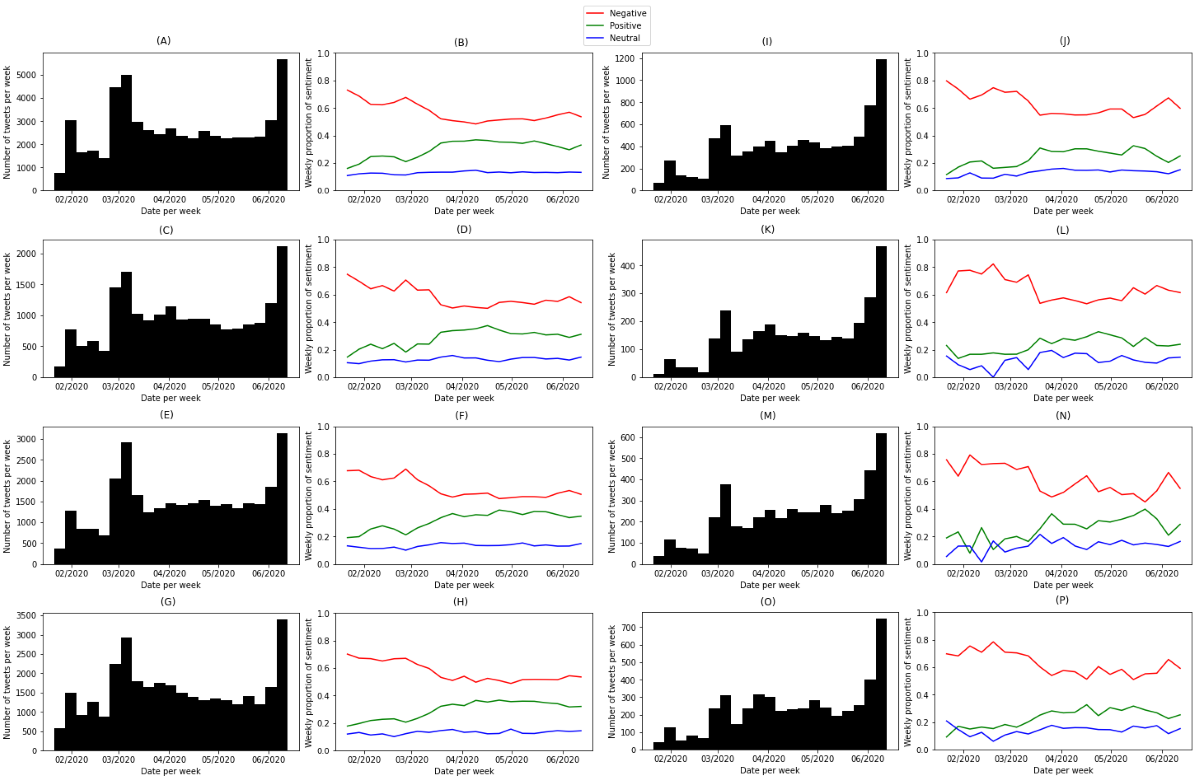
The number of tweets and proportion of sentiment, respectively, per week in the COVID-19 data set in (A) and (B) California, (C) and (D) Florida, (E) and (F) New York, (G) and (H) Texas and in the Centers for Disease Control and Prevention (CDC) subset split in (I) and (J) California, (K) and (L) Florida, (M) and (N) New York, (O) and (P) Texas.

Further, we chose 4 states with the highest volumes of tweets (ie, California, n=56,188; Texas, n=32,890; New York, n=31,178; and Florida, n=19,965) for temporal analysis. Figure 7 shows the volumes and sentiment polarity toward COVID-19 issues and preventive practices at the weekly level for the 4 states. The timelines of the 4 states demonstrated similar patterns and were close to the general trend in the United States. We observed state differences in many places. Florida (11,554/19,965, 57.9%) showed more negative sentiment to COVID-19 issues than the other 3 states: California (31,926/56,188, 56.8%), Texas (18,682/32,890, 56.8%), and New York (17,020/31,178, 54.6%). The starting point of Stage 4, when negative sentiment started to increase, came early in Florida (approximately late April 2020), while for New York and California, Stage 4 started in the middle of May 2020.

There were more variations in public sentiment toward preventive practices among the 4 states. Overall, there was a higher proportion of negative tweets in Florida (1916/3087, 62.1%) than in California (5284/8588, 61.5%), Texas (2991/4949, 60.4%), and New York (2850/4865, 58.6%) in the CDC subset. California and Florida shared similar trends, where the timeline started with high ratios of negative tweets, which lasted until the middle of March 2020 and stayed relatively low and increased at the later stage. New York was different in that the public sentiment to preventive practices seemed to vary greatly in the timeline. The proportion of negative sentiment decreased to almost 40% in the middle of March 2020 and started to increase to about 60%, then decreased to converge to almost 40% and increased at the later stage. There was a spike in negative sentiment in the middle of April 2020. After checking the term frequency-inverse document frequency (TFIDF) of keywords, we found keywords related to political figures, the Black Lives Matter movement, and various current events. This shows that topics were not solely related to COVID-19 nor preventative practices and tweets’ sentiments may have additional influence from other topics. In Texas, there was decreasing negative sentiment until the middle of April 2020, when there was a spike in negative sentiment. Following that, the negative sentiment increased gradually.

### Sentiment Polarity in Urban and Rural Areas

We conducted *t* tests to compare the public sentiment toward COVID-19 and preventive practices in the urban and rural areas of counties. The results are shown in Table 4. Counties were split into their respective urban and rural areas. After filtering urban and rural counties that did not have at least 15 tweets, 830 counties with urban areas and 182 counties with rural areas remained in the COVID-19 data set, and 355 urban and 52 rural counties remained in the CDC subset.

The *t* tests showed that there were no significant differences toward COVID-19–related issues. However, public discussions on preventive practices (CDC subset) were significantly more negative in rural areas (mean 0.6543, SD 0.0785) than in urban areas (mean 0.6112, SD 0.0891; t_405_=–3.6332, *P*<.001). Additionally, we observed more positive sentiment for people in urban (mean 0.2454, SD 0.0822) than in rural areas (mean 0.2173, SD 0.0716; t_405_=2.5976, *P*=.01) and a higher proportion of neutral posts in urban areas (mean 0.1433, SD 0.0565) than in rural areas (mean 0.1283, SD 0.0411; t_405_=2.313, *P*=.02).

**Table 4:** Comparison of sentiment polarity between urban and rural areas.

Sentiment	COVID-19 data set					CDC^a^ subset			
	Urban, mean (SD)	Rural, mean (SD)	*t* value	*P* value	Urban, mean (SD)		Rural, mean (SD)	*t* value	*P* value
Negative	0.5768 (0.0897)	0.5833 (0.1064)	–0.7691	.44	0.6112 (0.0891)		0.6543 (0.0785)	–3.6332	<.001
Positive	0.2921 (0.0827)	0.2918 (0.0995)	0.0397	.97	0.2454 (0.0822)		0.2173 (0.0716)	2.5976	.01
Neutral	0.1310 (0.0505)	0.1248 (0.0588)	1.3211	.19	0.1433 (0.0565)		0.1283 (0.0411)	2.3213	.02

^a^CDC: Centers for Disease Control and Prevention.

#### Sentiment Polarity and Socioeconomic Factors

The variances in public sentiment toward COVID-19 and preventive practices were then examined with the socioeconomic indicators of poverty and unemployment rates, as well as the median household income. The normality of these 3 variables and sentiment polarity was checked. Table 5 presents the distributions of all socioeconomic factors, the sentiment values, and the average tweet populations for counties.

Table 6 presents the Pearson correlation results. The unemployment rate was positively correlated with the proportion of negative sentiment (*r*_907_=0.0982, *P*=.003) and negatively correlated with the proportion of positive sentiment (*r*_907_=–0.1407, *P*<.001) in the COVID-19 data set. It means counties with higher unemployment rates had higher negative sentiment polarity toward COVID-19 issues. Similarly, counties with higher poverty rates tended to have a lower proportion of positive discussions on COVID-19 issues (*r*_907_=–0.0836, *P*=.01). Finally, median household income was negatively correlated with the proportion of negative sentiment (*r*_907_=–0.1322, *P*<.001) and positively correlated with the proportion of positive sentiment (*r*_907_=0.1554, *P*<.001) in the COVID-19 data set. No significant correlations were found between any socioeconomic factors and public sentiment toward preventive practices.

**Table 5:** Mean (SD) for all socioeconomic and sentiment variables.

Variable	COVID-19 data set (909 counties), mean (SD)	CDC^a^ subset (413 counties), mean (SD)
Number of tweets	371.9417 (1052.9472)	119.2421 (229.7825)
Poverty rate	12.4608 (4.6809)	11.8521 (4.5293)
Unemployment rate	3.7809 (1.2576)	3.6608 (1.2024)
Median household income (US $)	61489.36 (16394.21)	66516.33 (17888.07)
Proportion of negative sentiment	0.5769 (0.0877)	0.6118 (0.0886)
Proportion of positive sentiment	0.2922 (0.0823)	0.2446 (0.0817)
Proportion of neutral sentiment	0.1309 (0.0494)	0.1436 (0.0571)

^a^CDC: Centers for Disease Control and Prevention.

**Table 6:** Associations among socioeconomic factors and negative, positive, and neutral polarities.

Variable		COVID-19 data set			CDC^a^ subset	
		Coefficient	*P* value	Coefficient		*P* value
**Negative**						
	Poverty rate	0.0461	.17	–0.0261		.60
	Unemployment rate	0.0982	.003	0.0770		.12
	Household income	–0.1322	<.001	–0.0203		.68
**Positive**						
	Poverty rate	–0.0836	.01	–0.0039		.94
	Unemployment rate	–0.1407	<.001	–0.0661		.18
	Household income	0.1554	<.001	0.0329		.51
**Neutral**						
	Poverty rate	0.0574	.08	0.0460		.35
	Unemployment rate	0.0599	.07	–0.0249		.61
	Household income	–0.0242	.47	–0.0155		.75

^a^CDC: Centers for Disease Control and Prevention.

## Discussion

### Principal Findings

We conducted 4 types of analysis to answer the 4 research questions. The time series analysis revealed the 4 stages of change in public sentiment toward COVID-19 and the preventive practices for research question 1. People showed high negativity in the initial stage from late January 2020 to the beginning of March 2020, when the COVID-19 risks were not widely recognized in the United States. Wise et al [13] identified that the first week of the COVID-19 pandemic in the United States was March 11, 2020 to March 16, 2020. The first stage mainly reflected how the US population viewed COVID-19 in other countries. Starting from the week of March 11, 2020, when COVID-19 was identified as affecting the United States, people demonstrated growing awareness of the risks associated with COVID-19 and were more engaged in preventative behaviors [13]. Our findings, based on Twitter data, showed similar patterns in that people started to have fewer negative discussions on COVID-19 issues and showed more positive attitudes toward preventive practices in Stage 2. However, the decreasing trend in negative sentiment did not persist. In Stage 3, the proportion of negative sentiment remained stable and lasted for a month. After that, people started to show increasing negative sentiment to both COVID-19 issues and preventive practices, which was not a good sign at a time when the pandemic was far from over. These findings illustrated several challenges in the communication strategies of public health authorities and in government policy making. The first challenge is how to inform people about the disease and its potential risks as well as to convince people to take actions to prevent the spread of the virus in the initial stage when the risks are not geographically close. The second challenge is that people may change their attitudes toward preventive practices after they have experienced the pandemic and obtained information about the disease. It is important to understand what led to the change in their attitudes and behaviors and how long it takes for people to adapt to or get tired of the changing behaviors.

We analyzed the dynamics of public sentiment at the state level and presented the results from 4 states—California, Florida, New York, and Texas—which showed similar patterns but differed in the timing of the turning points and sentiment polarity to answer research question 2. Our findings were consistent with some existing studies. For example, Hung et al [31] found that Florida was one of the states that expressed the most negative sentiment in COVID-19–related discussions, and our study showed that Floridians were generally more negative in their discussions on general COVID-19 topics and preventive practices.

For research question 3, our study further revealed that people in rural areas generally have more negative sentiment toward COVID-19 issues and preventive practices suggested by the CDC. Czeisler et al [30] conducted representative panel surveys and found that people in New York City and Los Angeles, which are large urban clusters, had more agreement on the stay-at-home orders, business closures, self-isolation, and wearing facial masks in public than the general US population. These findings could be helpful in guiding public authorities in decision making and policy development, for example, to consider the urban and rural differences in communication strategies and guidance.

Further, median household income, as well as poverty and unemployment rates, were not associated with differences in public sentiment to preventive practices; however, higher unemployment rate was positively correlated with negative polarity to COVID-19–related topics, which addresses research question 4. The finding was different from that in the survey study by Czeisler et al [30], which showed people who were unemployed had more agreement on social distancing, wearing masks, stay-at-home orders, and business closures and were less likely to accept the reopening of the United States. The differences might be caused by the sampling method used in the survey. Combined with the results of the urban/rural analysis, we suggest that different policies or communication strategies may be considered more from the urban/rural perspective than based on socioeconomic differences in pandemics similar to COVID-19.

### Limitations

This study relied on geolocated Twitter data to estimate sentiment polarity at different levels of temporal and spatial granularity. We used the followers-to-followees ratio to remove accounts that were potentially nonindividual users such as bots, which may not be fully accurate. For future work, we believe a bot detection algorithm incorporating more user information may provide more accurate user filtering. Twitter users who have geolocated posts are profiled to be of the younger generation with higher socioeconomic levels who may not represent the whole population in the United States. Considering that the proportion of such users in the population is similar across counties or states, the comparative directions with sampled Twitter users can be representative. To avoid biased interpretation, our findings focused more on the directions and significant level of relationships rather than how large the differences or the correlation coefficients were. Studies with social media data are valuable as they could provide time-sensitive knowledge at different spatial scales, which are difficult to achieve with survey studies in a cost-effective way. Notably, survey methods are irreplaceable to collect attitudes of people who do not go online.

Another limitation came from the algorithm we used for the detection of sentiment. Although the pretrained deep learning model has state-of-the-art sentiment classification accuracy, it may generate wrong sentiment classifications for posts. When the data are scarce, the errors caused by the detection algorithm may lead to large variances in the aggregated sentiment polarity. That is why we adjusted the temporal granularity in the computation of public sentiment for preventive practices and for states. Given more scarce data in the study of other topics, the choice of aggregation level should be more coarsely grained.

Finally, we focused on the sentiment and classified posts as positive, negative, or neutral. There is a need for a deeper understanding and assessment of Twitter content to accurately characterize reaction in multiple dimensions, such as support, hope, and happy that belong to the positive sentiment and fear, despair, and hate that belong to the negative sentiment [32].

### Comparison With Prior Work

Many researchers have studied online discussions, specifically public sentiment, and popular topics, during COVID-19 for timely situational awareness. For example, Xiang et al [33] examined discussions related to older adults on Twitter between January 23, 2020 and May 20, 2020. They identified the lockdown theme was the most popular one where “fear” and “sadness” were the prevalent sentiments. Wang et al [12] analyzed the topics and associated sentiment of social media posts about COVID-19 in China. There were increasing negative emotions expressed from January 20, 2020. Worries about production activity, such as “go to work” and “resume work,” started to grow from January 26, 2020. In our study, we focused on topics related to preventative COVID-19 practices on Twitter. Although they have been studied locally with survey methods [13,30], few have systematically investigated the topics through social media analysis.

Studies have been done to analyze public sentiment from the perspectives of temporal variations and spatial disparities in COVID-19. Xi et al [34] used Weibo data to understand concerns of the elderly during COVID-19 in China. They identified 3 temporal stages from January 20, 2020 to April 28, 2020, with “older adults contributing to the community” in the first stage and “older patients in hospital” in the second and third stages. Zhou et al [6] tracked the sentiment dynamics of tweets on COVID-19 in Australia regarding topics such as lockdown and social distancing. The overall sentiment polarity toward these policies changed at different stages. Positive sentiment played a dominant role initially but decreased over time. Li et al [8] analyzed English tweets from March 25, 2020 to April 7, 2020. Their results showed a high variation in sadness, anger, and anticipation in tweets containing the term “mask” and disgust and sadness in tweets containing the term “lockdown.” A temporal analysis on COVID-19 tweets from January 2020 to June 2020 in 4 countries showed that negative sentiment increased following the lockdown policy enforced by the government of these countries [35].

Several studies have leveraged the geolocation information in social media data to examine public sentiment in different administrative units. Han et al [36] analyzed microblogs in China and showed that the topic “Government response” was the most prominent in Beijing, Sichuan, and Xi’an, while in the surrounding areas of Wuhan, negative sentiment and the topic “Seeking help” were trending in early 2020. Nilima et al [37] investigated the psychosocial factors associated with COVID-19 and the lockdown in India. They detected a clustering of places with similar reaction patterns and found people in different states have different concerns. Imran et al [38] found people’s reactions to COVID-19 were culturally different, as people in Pakistan and India showed different sentiment patterns from people in the United States and Canada. Not many studies have specifically examined the discussions in the United States. Van et al [29] investigated public attitudes to social distancing in the United States and found there were geographical variances, which can be partially explained by political ideologies. Chun et al [4] collected tweets in one week of March about government enforcement for the spreading of COVID-19 and calculated the citizens’ concern index for different measures. It showed that school closing–related tweets contained the highest levels of concern. Our findings contribute to the knowledge of public sentiment and public opinions related to COVID-19 on social media platforms in the United States. We conducted a comprehensive study to analyze temporal changes from the initial stage when COVID-19 was yet to spread in the United States to the stage when people started to show rebound in negative sentiment or resistance to preventive practices.

Additionally, we explored the association between public sentiment polarity and other geographical and socioeconomic factors to identify factors that were related to the spatial disparities. The findings could be helpful to guide public health authorities in decision making and policy development in a similar pandemic in the future.

In this study, we applied a data analysis framework to investigate public sentiment toward COVID-19 and the preventive practices suggested by public health authorities in the United States. The data processing framework can be applied to the analysis of discussions on other topics such as vaccination and reopening evaluation in COVID-19 or provide useful solutions for future crises.

### Conclusions

This study used a data-driven method to understand public sentiment to the COVID-19 issues and preventive practices with geolocated Twitter data. We first used a deep learning model to acquire the sentiment of each tweet. These tweets were then aggregated into different temporal and geographical units to measure the polarity of public sentiment.

In the temporal analysis, we discovered 4 stages of change that were evident in discussions on both COVID-19 issues and preventive practices, demonstrating a common pattern between the 2 topics. Based on the examination of our sample of 4 states with the largest volume of tweets across the time period studied, Florida had more negative sentiment to COVID 19 issues and CDC preventive practices than California, Texas, and New York. We analyzed the spatial disparities and explored whether the variations in public sentiment were associated with geographical factors and discovered that there were significant differences in sentiment polarity to preventive practices between urban and rural areas. Socioeconomic factors such as median household income, as well as poverty and unemployment rates, were significantly related to sentiment polarity to COVID-19 issues but not to preventive practices.

The insight gained from the study could be helpful for public health authorities and governments to adjust and differentiate the communication strategies and policies throughout the stages of a pandemic. Communication strategies and policies should be considered based on urban and rural differences more than socioeconomic differences.

## Abbreviations

API: application programming interface
CDC: Centers for Disease Control and Prevention
PPE: personal protective equipment
TFIDF: term frequency-inverse document frequency
